# Conservation and functional influence of alternative splicing in wood formation of *Populus* and *Eucalyptus*

**DOI:** 10.1186/1471-2164-15-780

**Published:** 2014-09-10

**Authors:** Peng Xu, Yimeng Kong, Dongliang Song, Cheng Huang, Xuan Li, Laigeng Li

**Affiliations:** National Key Laboratory of Plant Molecular Genetics and Key Laboratory of Synthetic Biology, Institute of Plant Physiology and Ecology, Chinese Academy of Sciences, 300 Fenglin Rd, Shanghai, 200032 China

**Keywords:** Alternative splicing (AS), Comparative transcriptome, Wood formation, Conservation, *Populus*, *Eucalyptus*

## Abstract

**Background:**

Wood formation in tree species is regulated by multiple factors at various layers. Alternative splicing (AS) occurs within a large number of genes in wood formation. However, the functional implications and conservation of the AS occurrence are not well understood.

**Results:**

In this study, we profiled AS events in wood-forming tissues of *Populus* and *Eucalyptus*, and analyzed their functional implications as well as inter-species conservation. 28.3% and 20.7% of highly expressed transcripts in the developing xylem of *Populus* and *Eucalyptus* respectively were affected by AS events. Around 42% of the AS events resulted in changes to the original reading frame. 25.0% (in *Populus*) and 26.8% (in *Eucalyptus*) of the AS events may cause protein domain modification. In the process of wood formation, about 28% of AS-occurring genes were putative orthologs and 71 conserved AS events were identified in the two species.

**Conclusion:**

Through analysis of AS events in developing xylem of two tree species, this study reveals an array of new information regarding AS occurrence and function in tree development.

**Electronic supplementary material:**

The online version of this article (doi:10.1186/1471-2164-15-780) contains supplementary material, which is available to authorized users.

## Background

Alternative splicing (AS), which generates multiple transcript variants from one gene, is a key scheme in multicellular eukaryotes to enhance the functional diversity of the proteome [1]. In flowering plant species, up to 61% of multiexonic genes in *Arabidopsis* and 48% of genes in rice are affected by AS [2, 3]. AS has been shown to regulate plant development in photosynthesis, disease resistance, floral transition, circadian rhythm, starch metabolism, auxin biosynthesis, and temperature response [4–6]. AS can cause alterations to protein function through modulating protein structure. For example, frame shifts caused by AS often result in truncated proteins that can form nonfunctional heterodimers and act as dominant-negative regulators [7, 8]. AS occurrence in functional domain structures can affect protein-protein interaction, transcriptional activation or DNA binding [9–11]. In addition to modulate protein functions, AS can influence transcript stability through nonsense-mediated decay (NMD) or miRNA linked regulation [12, 13].

Wood, a unique structural and storage tissue in tree species, is derived from the meristematic activities of the vascular cambium during secondary growth [14, 15]. Wood formation involves a coordinated progression of cell differentiation, expansion, secondary cell wall formation, and programmed cell death [16]. AS has been found to play important roles in the process of wood formation. NAC transcription factor *PtrWND1B* / *PtrSND1-A2,* which controls secondary wall biosynthesis, was shown to undergo AS in *Populus*[17, 18]. The splice variant of *PtrWND1B/PtrSND1-A2*^*IR*^ contains a retained intron and encodes a protein lacking DNA binding and transactivation activity but retaining dimerization ability. PtrSND1-A2^IR^ functions as a dominant negative of PtrSND1 members through heterodimerization [17]. This dominant negative regulation was demonstrated as a specific mechanism controlling fiber cell wall thickening during wood formation in *Populus*[18]. AS may have evolved as a regulatory mechanism of the isochorismate synthase gene, *PtiICS*, which encodes an enzyme for phylloquinone biosynthesis, in *Populus*[19].

Analyzing how AS is conserved across species can help to identify AS events important in general for plant development. Conserved AS events in several gene families such as ribulosebisphosphate carboxylase/oxygenase activase, SR protein, MYB transcription factor and transthyretin-like protein have been characterized in plant species [20–23]. Genome-wide analysis have identified 56 conserved AS events between *Arabidopsis* and rice, and 49 conserved AS events between rice and maize [24]. Between two or more legume species, 22 conserved AS events were detected [25]. More conserved AS events (527 cases) were reported between closely related species *Brassica* and *Arabidopsis*[26]. Meanwhile, it is not known how AS events are conserved and involved in modulating protein function in wood formation of tree species.

Recently, transcriptome of *Populus* xylem was analyzed to identify AS events in wood formation tissue. Up to 36% of expressed genes were detected to undergo AS [27], indicating a wide influence of AS in the process of wood formation. On the other hand, great variations of AS events, which are likely caused by genotypic polymorphism, were observed among different *Populus* populations [27]. Although AS has shown to influence diverse groups of genes in *Populus*, how the AS events are involved in protein function modulation is little known. In this study, we compared the occurrence of AS in *Eucalyptus* and *Populus* and analyzed the functional implications and conservation of AS during wood formation in tree species.

## Results

### AS profiles in wood formation tissues

To detect AS events during wood formation, we first constructed transcriptomes of developing xylem in *Populus* (*P. × euramericana*) and *Eucalyptus* (*E. grandis*). Two biological replicates were sampled from the two species for transcriptome analysis using the Illumina platform based RNA-seq technology [28]. The sequencing analysis generated approximate 30 ~ 45 million paired-end reads (100-nucleotide length) from each sample of the two species. 77.6% ~ 80.6% of these reads could be mapped onto the reference genome via the TopHat program [29]. More than 60% of the mapped reads located in the exon regions of the two genomes (Additional file 1). In *Populus*, 26.2% of the reads were mapped to an exon-intron/intron-exon junction region, among which 21.3% were not annotated in the reference genome. This part of reads could be from the products caused by AS variants. In *Eucalyptus*, 36.8% of the junction reads had no annotations. Some reads were found to map to intergenic regions. 6.8% of the total reads in *Eucalyptus* were mapped to the intergenic regions of the genome while 0.9% of reads in *Populus* showed such mapping. This may reflect the genome annotation difference in two species.

Expressed transcripts were assembled by the Cufflinks program with the cutoff of 0.1 FPKM in both biological replicates [30]. Considering that the weakly expressed transcripts could result in false AS identification [2], highly expressed transcripts with an expression level higher than 5% of the most abundant transcript were subsequently used as the basis for follow up analysis. In *Populus*, 31,984 highly expressed transcripts were assembled. Among them, 13.8% (4,397) of transcripts had no annotation in the reference genome (Additional file 1). In *Eucalyptus*, the number of highly expressed transcripts was 21,372 and 19.5% of them were not annotated in the genome. The assembled transcripts were transcribed from 23,735 (57.4% of the total predicted genes) genes in the *Populus* genome and 17,202 (47.3% of the total predicted genes) genes in the *Eucalyptus* genome (Additional files 1 and 2), suggesting that a large portion of the genes in the two species are expressed in developing xylem tissue.

**Figure 1:**
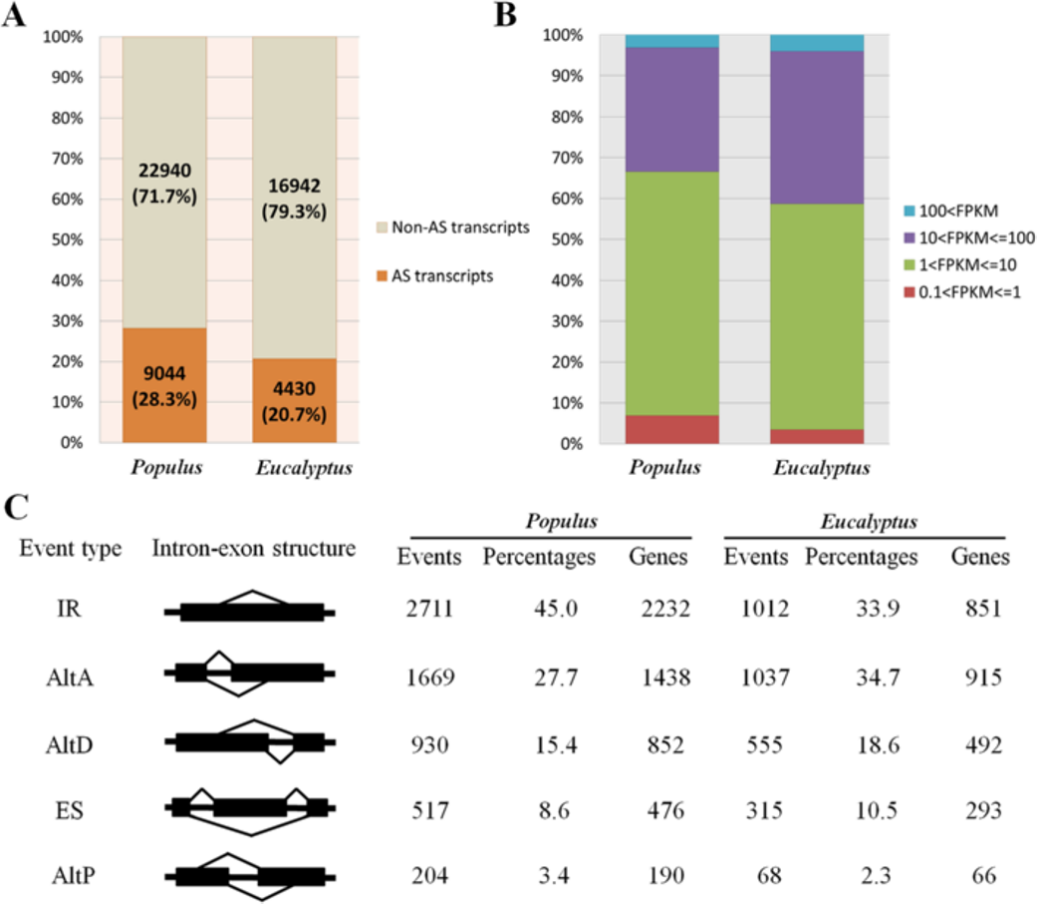
**AS profiles in developing xylem of** ***Populus*** **and** ***Eucalyptus*** **. (A)** Percentages of expressed transcripts undergoing AS in two species. **(B)** Expression levels of AS transcript isoforms. The expression abundance was measured with unit of FPKM (Fragments Per Kilobase of transcript per Million mapped reads). **(C)** Distribution of different AS types. IR, intron retention; AltA, alternative acceptor site; AltD, alternative donor site; ES, exon skipping; AltP, alternative position.

On the basis of the assembled transcripts, AS events among the transcriptome was analyzed. The ASTALAVISTA tool [31] was used to establish the repertoire of AS events. In total, 6,031 AS events in *Populus* and 2,987 events in *Eucalyptus* were detected in developing xylem tissue (Additional file 3). These AS events affected 28.3% and 20.7% of the highly expressed transcripts in the two species (Figure 1A), respectively, which were transcribed from 17.2% (4,079 in *Populus*) and 11.9% (2,039 in *Eucalyptus*) of the xylem expressed genes. The majority of AS transcripts (93% in *Populus* and 97% in *Eucalyptus*) displayed expression levels higher than 1.0 FPKM (Figure 1B). Over 85% of the AS events occurred in the transcript isoforms with expression ratios of 0.1 ~ 1.0 compared to the major transcript of a same gene (Additional file 4). For verification of the AS events, 16 representative AS events were selected for RT-PCR analysis and 14 of them (about 87.5%) were confirmed (Additional file 5), indicating the reliability of the AS detection in our RNA-seq analysis.

The constructed AS events were categorized into five major AS types [32]: intron retention (IR), alternative acceptor site (AltA), alternative donor site (AltD), exon skipping (ES) and alternative position (AltP) for further analysis. IR, which encompassed 45.0% and 33.9% of the AS events in *Populus* and *Eucalyptus* respectively (Figure 1C), represented a much higher portion of AS events than ES. AltA was over-represented and IR under-represented while the proportions of the other three AS types were similar in *Eucalyptus* compared to *Populus* (Figure 1C).

### AS features in wood formation

**Figure 2:**
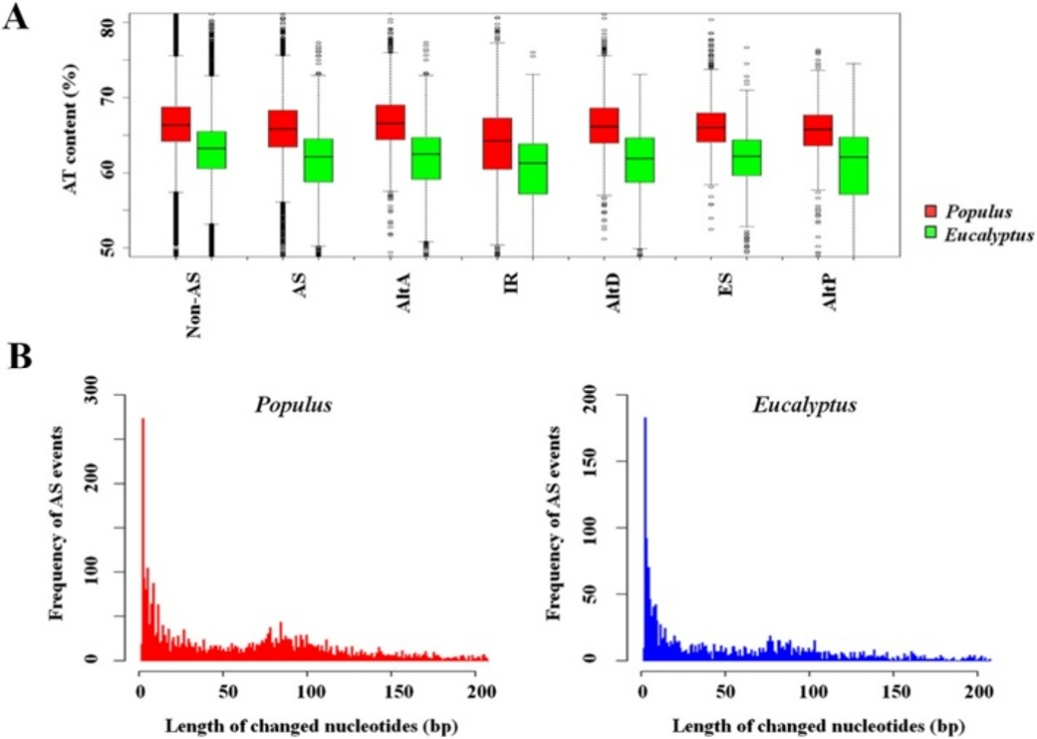
**AS profile features in developing xylem of** ***Populus*** **and** ***Eucalyptus.*** **(A)** Comparison of AT content in constitutive introns (non-AS introns) and different types of alternative introns (AS introns). IR, intron retention; AltA, alternative acceptor site; AltD, alternative donor site; ES, exon skipping; AltP, alternative position. **(B)** Histogram indicating frequency of ORF located AS events at different changed lengths of nucleotides.

Based on the identified AS events in *Populus* and *Eucalyptus*, AS features in woody tissues were analyzed, including AT content of the introns, and nucleotide change caused by AS. In *Populus*, the average AT content of the total introns of xylem expressed genes is 66.4%, higher than 62.4% in *Eucalyptus* (*p*-value < 0.0001, *t*-test). Similarly, the average AT content across all alternative introns (AS introns) in *Populus* was 65.6%, higher than that in *Eucalyptus* (61.1%) (Figure 2A). This difference between species was also consistent in the introns among various AS types. Nevertheless, the nucleotide composition at splicing junction site was conserved between *Populus* and *Eucalyptus* (Additional file 6).

Selection of different splice sites may result in a string of nucleotides changed between transcript variants. The changed nucleotides could interrupt the original reading frame if they are not multiples of 3 base pairs. About 67% of the AS events were found to be located in the open reading frame (ORF) region of the two species. Analysis of ORF located AS events revealed that 79% of the changed nucleotides were between 1 and 200 bps (Figure 2B). A change of 3 nucleotides occurred most frequently in both *Populus* and *Eucalyptus*. Similar phenomenon was reported in *Arabidopsis*, rice and *Brassica*[26, 33]. The 3 nucleotide change was mainly generated from AltA, AltD and AltP, while IR or ES largely caused changes of 50-150 bp (Additional file 7). We observed 37% of the changed lengths fitting in multiples of 3 nucleotides in the two species, which could maintain the original reading frame. In other words, up to 63% of ORF located AS events (42% of the total AS events) in the two species could cause frameshift. A similar frequency of ORF change was observed in the AS isoforms with different expression ratios (Additional file 4).

### Protein domain modification caused by AS

**Figure 3:**
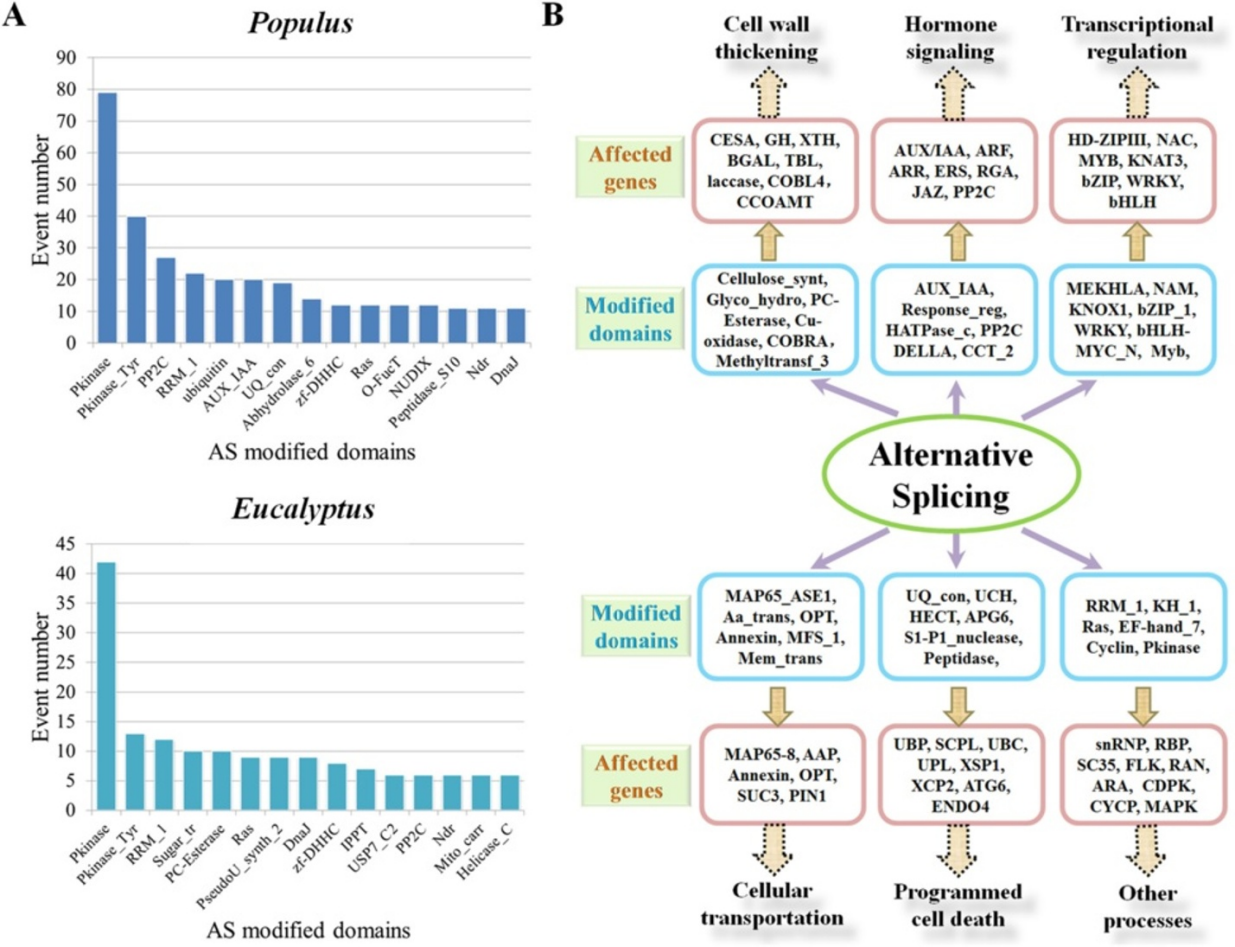
**Influence of AS on protein domains during wood formation. (A)** The 15 most frequently modified domains in *Populus* and *Eucalyptus*. Protein domains were identified from the Pfam database with the E-value cutoff of 1e-10. **(B)** Association of AS modified domains, AS affected genes and their potentially involved biological processes.

One of the major effects of AS is to create protein isoforms that increase proteome diversity [5, 34]. Previous studies suggest that protein functions could be affected if their functional domains are interrupted by AS [9–11, 17, 18]. We conducted *in silico* transcript translation and then analyzed whether AS occurrence caused protein domain alteration. In the two species, 25.0% (1507 events in *Populus*) and 26.8% (801 events in *Eucalyptus*) of the AS events occurred within domain sequences which would cause domain modification (Additional file 8). A similar frequency of such domain modification was observed in the AS isoforms with different expression ratios (Additional file 4). In detected AS events, 789 and 500 kinds of domains might be modified in *Populus* and *Eucalyptus*, respectively. Moreover, 16.6% (1003 events in *Populus*) and 18.2% (545 events in *Eucalyptus*) of AS events could result in domain gain or loss. Among the 15 most frequently modified domains, 8 domains were present in both *Populus* and *Eucalyptus*, namely Pkinase, Pkinase_Tyr, RRM_1, PP2C, zf-DHHC, Ras, Ndr and DnaJ (Figure 3A). To exclude the potential influence of domain number in the background genome, Fisher's exact test was used to obtain the enriched domains in the AS affected genes (Additional file 9). 51 enriched domains showed a close association with AS modification in the two species (*p*-value < 0.05), including domains RRM_1, PP2C and Ndr which were identified among the most frequently modified domains. This suggests that these protein domains may be particularly modified by AS events in wood formation process.

To understand how AS impacts wood formation, the AS modified domains from *Populus* and *Eucalyptus* were mapped to individual genes associated with wood formation according to their annotation (Additional file 10). These genes could be categorized into five fundamental processes for wood formation including: cell wall formation, hormone signaling, transcriptional regulation, cellular transportation and programmed cell death (Figure 3B). For example, cellulose synthase (CESA) and caffeoyl-CoA 3-O-methyltransferase (CCOAMT) are involved in secondary cell wall thickening during wood formation [35–37]. The occurrence of AS was observed in the catalytic domains of these enzymes. The functional domains of many other wood formation-related genes was also detected to be affected by AS.

### Conservation of AS-occurring genes in wood formation

**Figure 4:**
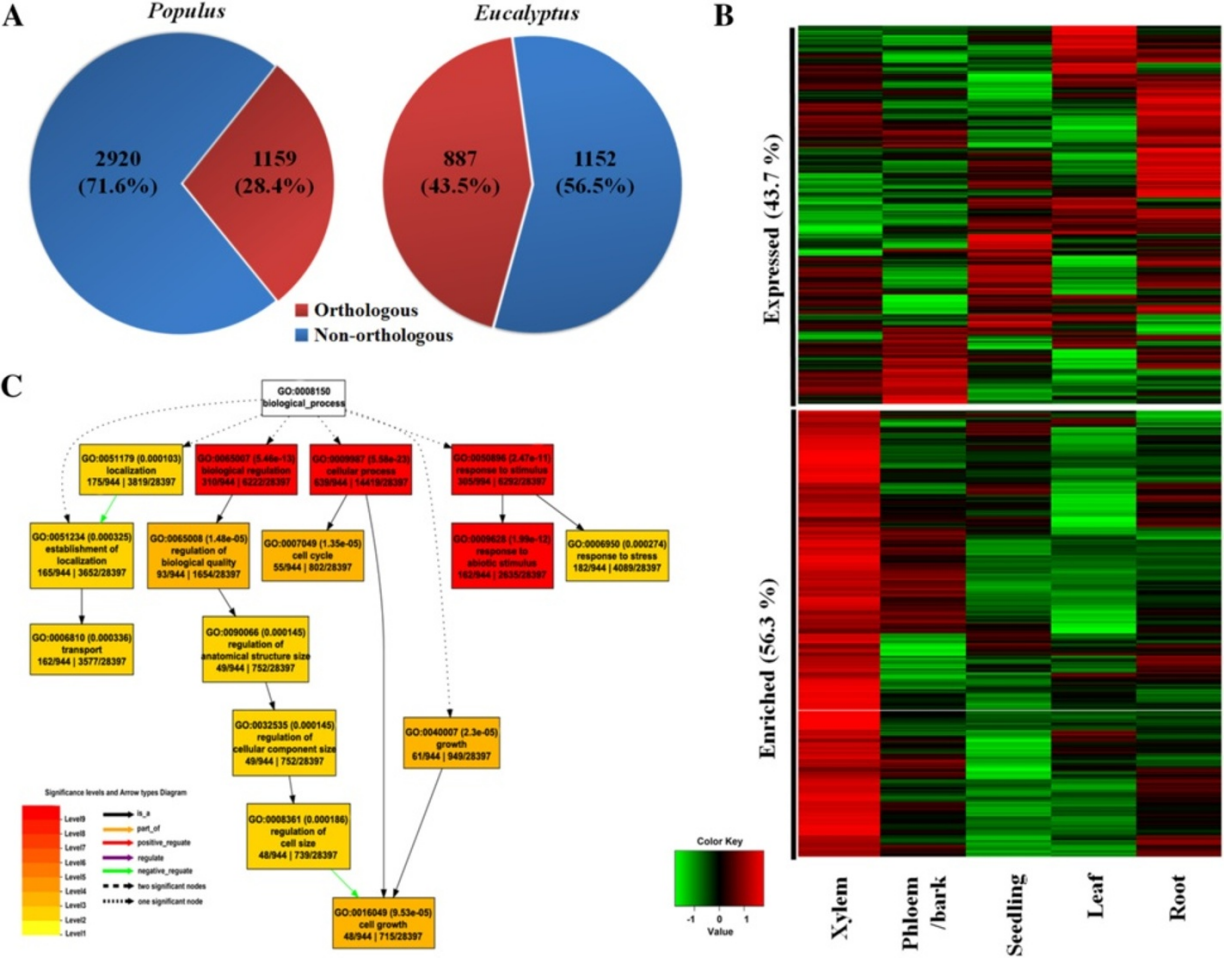
**Othologous genes affected by AS during wood formation. (A)** Orthology of AS-occurring genes in the xylem of *Populus* and *Eucalyptus*. **(B)** Tissue expression patterns of orthologous AS-occurring genes in *Populus*. Enriched genes showed the highest expression level in xylem tissue. **(C)** Enriched GO processes of orthologous AS-occurring genes in *Populus* (FDR < 0.001).

To investigate conservation of AS-occurring genes, putative orthologous genes in *Populus* and *Eucalyptus* were searched by the BLAST program (E-value < 1e-50, identity > 0.4 and coverage > 0.6). A total of 1159 *Populus* and 887 *Eucalyptus* putative orthologous genes were identified with AS-occurrence, which represents 28% and 44% of the AS-occurring genes in the two species, respectively (Figure 4A). Gene expression patterns established via microarray analysis [38, 39] revealed that 56.3% of these orthologous genes were highly expressed in xylem (Figure 4B), indicating their association with wood formation. These orthologs in the two species constituted 716 independent gene groups with different biological functions (Additional file 11). Gene ontology (GO) annotations suggested several important processes for plant development such as transportation, cell cycle and cell growth were significantly enriched (FDR < 0.001) (Figure 4C). Genes involved in the process responding to abiotic stimulus were also enriched, highlighting the potential impact of environmental cues on AS-occurring genes.

### Conserved AS events in wood formation

Among orthologous genes with AS occurrence, conserved introns were searched to identify conserved AS events across the two species. A total of 71 conserved AS events (1.2% of total AS events in *Populus* and 2.4% in *Eucalyptus*) were identified. These conserved AS events occurred between 65 *Populus* and 55 *Eucalyptus* genes (Additional file 12). Among them, 37 events belonged to the AltA type, ranking it as the most frequent type of AS. Approximately, 60% of the conserved AS events caused frameshift and 48% of the conserved AS events were detected to cause modification of functional domain structures (Additional file 12).

**Figure 5:**
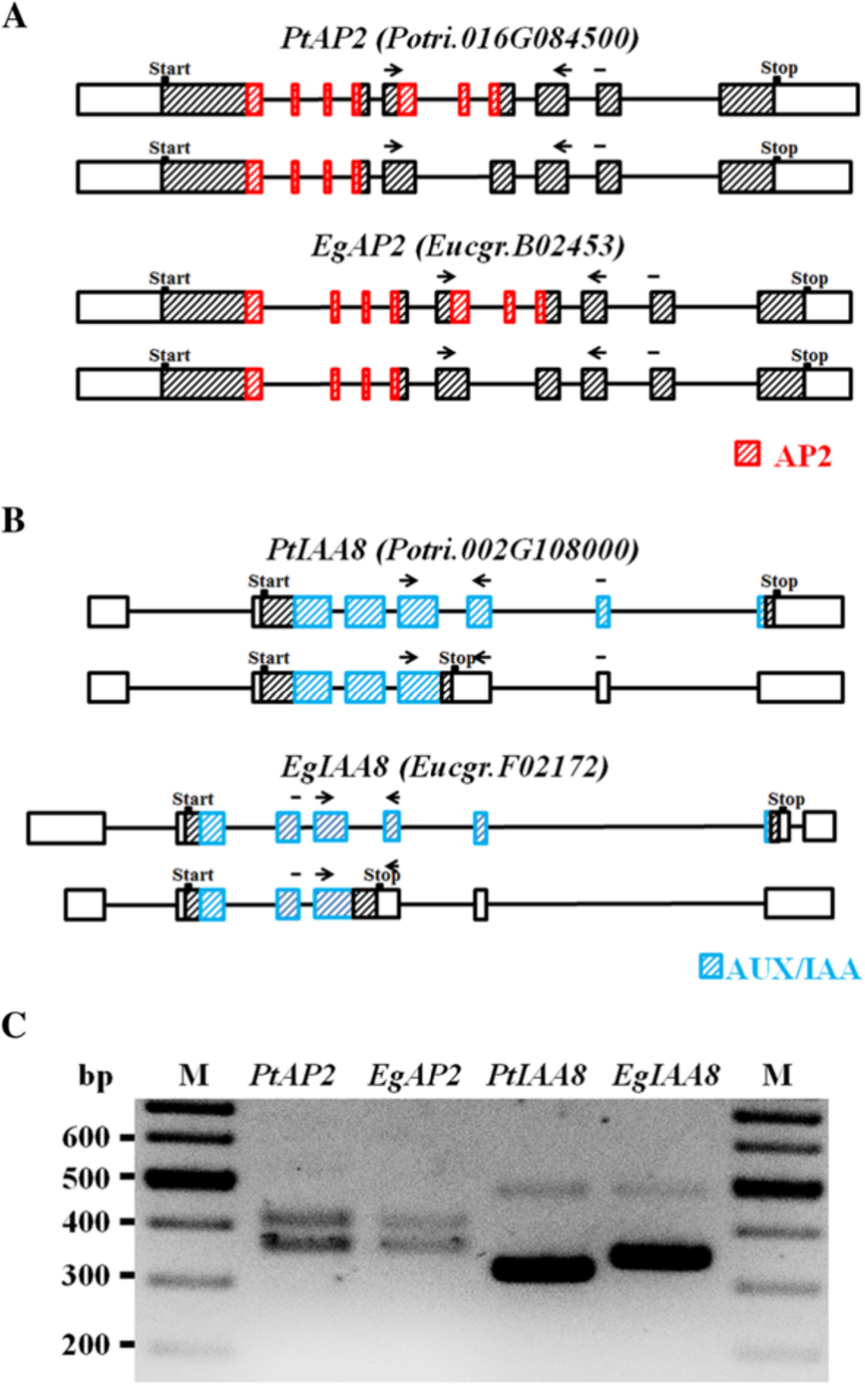
**Characterization of two conserved AS events in developing xylem of** ***Populus*** **and** ***Eucalyptus.*** Gene structures are shown for the conserved AS events in AP2 family **(A)** and AUX/IAA family **(B)**. Boxes represent exons and lines represent introns. Hatched regions indicate coding sequences. Red and blue colors indicate predicted domains. Primers designed for RT-PCR are shown by black arrows. **(C)** RT-PCR verification of the two conserved AS events. The upper bands indicate the full transcripts in *AP2* lanes and the intron-retained transcripts in *AUX/IAA* lanes.

Two examples of the conserved AS events in the two species were further analyzed in greater detail. AS was observed in an orthologous pair of AP2 genes in *Populus* and *Eucalyptus* (Figure 5A). The AP2/EREBP family encodes transcription factors that play a role in a variety of plant regulatory processes [40]. The AP2 domain of this gene family consists of 60 amino acids that are responsible for DNA binding. In *Populus*, one isoform of the *PtAP2* transcript has two AP2 domains while the second isoform only has one AP2 domain due to a skipped exon. The conserved AS event was also observed in *Eucalyptus*. Another example of conserved AS event occurred in the functional domain of Aux/IAA gene family, which encode short-lived nuclear proteins that negatively regulate auxin signaling transduction [41]. Aux/IAA proteins are likely involved in the early response to auxin signaling, which controls the development of wood-forming tissues [42]. Based on our analysis, a conserved intron is retained in the *IAA8* genes of *Populus* and *Eucalyptus*. Importantly, the conserved IR event resulted in a truncated protein as it introduced a premature termination codon in the transcript (Figure 5B). RT-PCR verified the two cases of conserved AS events in the two species (Figure 5C).

**Figure 6:**
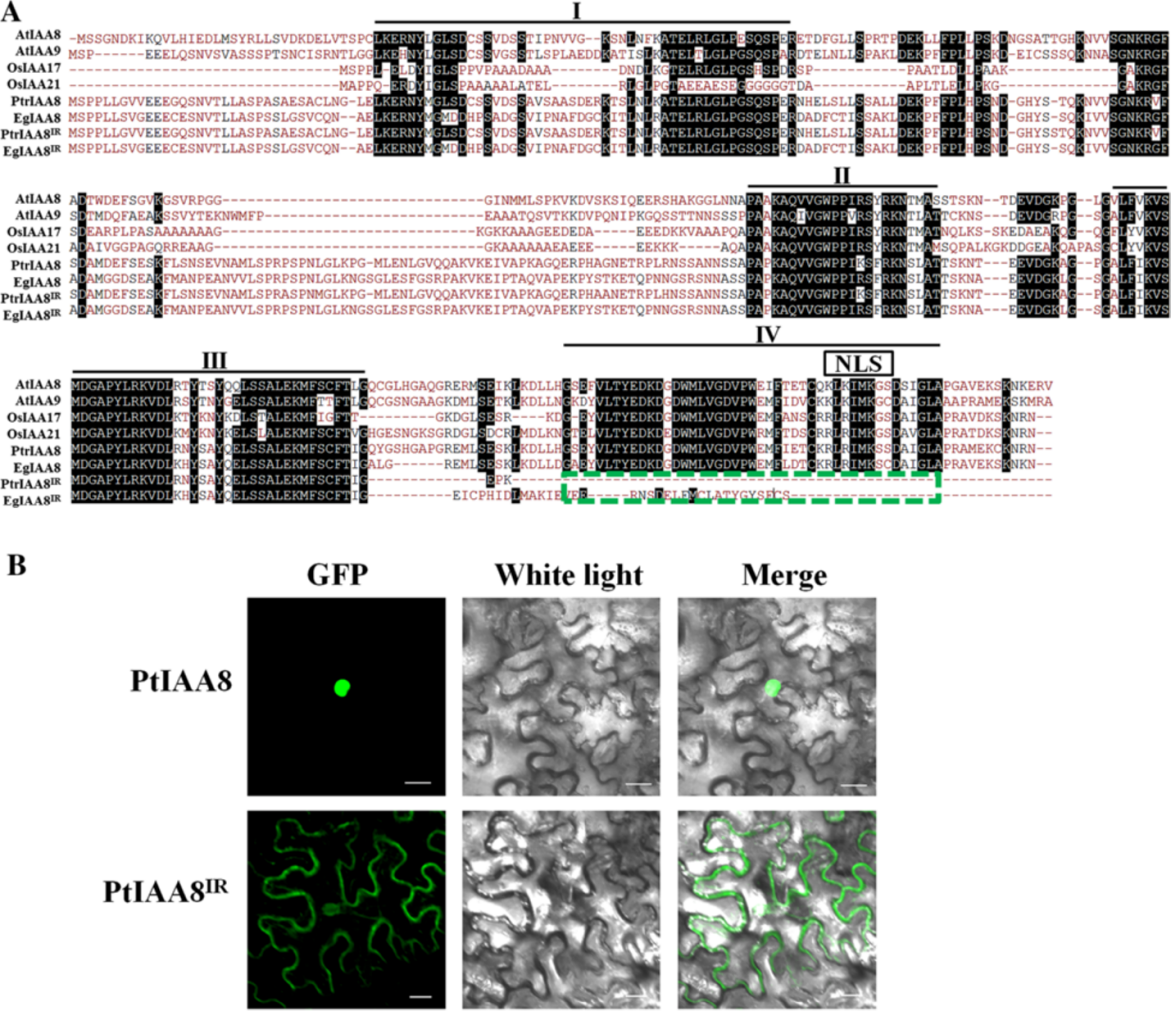
**Regulation of subcellular locations of IAA8 proteins by a conserved AS event. (A)** Protein sequence alignment of IAA8 orthologs in *Arabidopsis*, rice, *Populus* and *Eucalyptus*. Nuclear localization sequence (NLS) and four subdomains are shown on the top. Green box indicates the domain lost by the conserved IR event in *Populus* and *Eucalyptus*. **(B)** Subcellular localization of PtIAA8 isoforms. GFP fused PtIAA8 and PtIAA8^IR^ were transiently expressed in tobacco leaves and examined by confocal microscope. Bar: 20 μm.

To study how AS can regulate the function of the Aux/IAA family, sequences of IAA8 orthologs from *Arabidopsis*, rice, *Populus* and *Eucalyptus* were aligned. Multiple sequence alignment showed the truncated proteins, PtIAA8^IR^ and EgIAA8^IR^, specifically lack the conserved subdomain IV of Aux/IAA proteins, but contain intact subdomains I ~ III (Figure 6A). Subdomain IV contains a sequence which is critical for nucleus localization [43]. To examine whether the location of the protein is affected by AS, IAA8 isoforms from *Populus* were fused with GFP for localization analysis. When expressed in tobacco leaves, the protein encoded by full transcript PtIAA8 was shown to locate in the nucleus, while the truncated protein PtIAA8^IR^ displayed ubiquitous location in cells (Figure 6B). This indicates AS can modulate protein functions of Aux/IAAs through regulating their intercellular location.

## Discussion

### Landscape of AS in wood formation of tree species

This study established a comprehensive profile of AS events during wood formation. The results show that in both *Populus* and *Eucalyptus* more than 20% of the highly expressed transcripts were affected by AS, indicating extensive influence of AS on wood formation. In *Populus*, 6,031 AS events were detected while 2,987 events were detected in *Eucalyptus*. Based on current information, however, it is unknown whether this difference is implicated by the characteristics of the species. Five AS types were detected but their composition ratio was different in the two species. AltA was more frequently in *Eucalyptus* while IR was observed with a relatively greater frequency in *Populus*. Interestingly, intron sequences in *Eucalyptus* have a significantly lower AT content compared to those in *Populus*. Variations of AT content may be responsible for the different AS profiles in the two species, as UA-richness of plant introns is important for splicing efficiency [44]. AS identification in the developing xylem of *Populus trichocarpa* has recently reported [27]. About 7 ~ 11% of the total annotated genes were found to undergo AS in 20 *Populus* individuals from different populations, while around 46% of the AS events were not conserved within individuals, which may be caused by genotypic variations [27]. In the present study, the occurrence of AS was detected in about 10% of the total annotated genes and about 40% of the AS-occurring genes were the same in the reported individuals (PT17 and PT18) [27], indicating a consistency of AS occurrence in different *Populus* species. The results may also reflect some of the detected AS events being specific to the gene type of hybrid poplars (*P. × euramericana cv.‘Nanlin895’*).

### Functional implications of AS in wood formation

AS occurs extensively in various plant species, however, comprehensive analysis of their functional impact has been little studied. AS affect protein functions in a variety of ways by altering amino acid composition, subcellular location, secondary structure stability, binding property, and posttranslational modification [45]. The diversified aspects of AS influence can be assessed through *in silico* analysis. In this study, we primarily focused on analysis of AS-occurring genes which bring about modification of domain structures. Results show that about 42% of the AS events caused ORF disruptions or change, suggesting that AS extensively affects protein sequence. A large proportion (25.0% in *Populus* and 26.8% in *Eucalyptus*) of AS events resulted in protein domain modifications, which may be involved in wood formation at various levels. For example, the protein domains modified included Cellulose_synt, Glyco_transf and Glyco_hydro, which function in cell wall biosynthesis. Protein domains like proteasome and peptidase are involved in programmed cell death. AS modification to the AUX/IAA and PP2C domains indicates that AS may play a role in auxin and ABA signaling in wood formation tissue. We also observed a similar pattern of ORF change or domain modification in the AS events with different isoform ratios.

Apart from generating functionally tailored proteins, AS could also affect mRNA transport, localization and stability prior to protein translation. For example, some intron-containing transcript variants could remain within the nucleus resulting in decreased mobility [46]. This indicates a subset of AS transcripts which cannot be translated into proteins as *in silico* predictions would suggest. In addition, aberrant transcripts caused by AS are often degraded by RNA surveillance pathways such as nonsense-mediated decay (NMD) [12]. In theory, NMD sensitive transcripts would generally display low expression levels. A group of such AS events might be excluded from this study as weakly expressed transcripts were filtered out in analysis. Thus, a normalized cDNA library should be constructed in order to estimate NMD targets during wood formation [2]. Intriguingly, typical NMD targets in *Arabidopsis* include mRNA with a 3'UTR sequence longer than 350 bp [47–49]. Our analysis suggests that the average length of 3'UTR in both *Populus* and *Eucalyptus* is over 400 bp. And 3'UTR sequences are usually longer than 700 bp when they contained AS events. This may suggest a difference between *Arabidopsis* and tree species in the precise length of 3'UTR that could trigger NMD pathway. Taken together, our results indicate that the AS events may function in regulating wood formation through modifying protein domain structures.

### Conservation of AS in *Populus*and *Eucalyptus*

By comparing AS profiles in *Populus* and *Eucalyptus*, this study provides a sketch of AS events conserved in wood formation tissue. In our results, AS occurred within 1159 *Populus* and 887 *Eucalyptus* putative orthologous genes. GO analysis revealed that the orthologous genes were featured with functions related to the regulation of cell growth and differentiation. Over half of the orthologous genes were preferentially expressed in the developing xylem of *Populus*, indicating their involvement in wood formation.

Comparison of AS events identified 71 AS events that were conserved in *Populus* and *Eucalyptus*. Among them, 47.9% of the conserved AS events caused domain modifications. For instance, a conserved IR event was demonstrated to affect subdomain IV of Aux/IAA isoforms, which is critical to regulate protein subcellular location. The intron-retained *Aux/IAA* isoforms contain a premature termination codon and have a long 3’UTR sequence (>700 bp), suggesting they may be regulated by the NMD pathway that is coupled with AS occurrence. Consistent with this, these transcripts displayed reduced expression level according to RT-PCR. Interestingly, no equivalent AS regulation has been reported for *Aux*/*IAA* family genes in other plant species such as *Arabidopsis* and rice. Whether this AS event regulation of *Aux*/*IAA* family genes is specific to wood formation is yet to be further characterized.

## Conclusions

AS plays a critical role in the development of multicellular organisms, though much remains to be learned about its function in tree-specific processes. AS is extensively involved in wood formation as revealed by profiles of AS-occurring genes, AS events and AS modification of protein structures in the developing xylem of *Populus* and *Eucalyptus*. The results of this study highlight a number of new avenues to explore how AS is employed as a mechanism to modulate gene functions in wood-forming tissues.

## Methods

### RNA-seq experiment

The *Populus* (*P. × euramericana cv.‘Nanlin895’*) and *Eucalyptus* (*E. grandis*) were grown in experimental fields in Shanghai (31°N) and Guangxi Province (21°N). Developing xylem from 3-year old trees was sampled at mid-day (June, 2012) and razor blades were used to collect xylem tissues after peeling off the bark. Xylem tissues from three individual trees of each species were mixed together for sample preparation. Two biological replicates of xylem samples in each species were used for RNA extraction following the CTAB method. RNA concentration and quality were determined by the Agilent Bioanalyzer 2100. The cDNA library was constructed by polyA enriched RNA. 200-300 bp fragments were selected for non-directional 100 nucleotide paired-end sequencing, which was performed on an Illumina HiSeq 2000 according to Illumina protocols (San Diego, CA, USA).

### Detection of AS events

The reads from two biological replicates in each species were mapped and assembled independently. Reference genomes of *Populus* (v3.0) [50] and *Eucalyptus* (v1.1) [51] were obtained from Phytozome database. RNA-seq reads were mapped onto reference genomes via TopHat (v2.0.10) [29]. The maximum intron length was set to 6000 bp and other parameters were left as defaults. Unique mapped reads were extracted by samtools (v0.1.18) with a MAPQ cutoff of 20 [52]. Cufflinks (v2.1.1) was used for transcript assembly with supplied genome annotations [30]. Expression abundance of assembled transcripts was obtained from the Cufflinks output, which was measured in the unit of FPKM (Fragments Per Kilobase of transcript per Million mapped reads). Expression of 99% of the transcripts were consistent between two replicates in each species (FDR < 0.05), indicating a good reproducibility. Downstream analysis are based on the genes that could be detected by RNA-seq (>0.1 FPKM) in both biological replicates. To ensure the reliability of AS detection, weakly expressed transcripts with expression levels less than 5% of the most abundant transcript format from a gene, were filtered out before AS detection. Transcripts that remained in both replicates were subjected to ASTALAVISTA web server to discover AS events [31]. AS transcripts were extracted from each AS event and the percentage of AS transcripts was calculated through dividing the number of AS transcripts by that of the total transcripts. Within a gene, isoform ratio of the AS event was set as the expression level of the minor transcript divided by that of the most abundant transcript. Five major types of AS previously described in Wang *et al*. 2006 [32], including intron retention (IR), alternative acceptor site (AltA), alternative donor site (AltD), exon skipping (ES) and alternative position (AltP), were extracted for further analysis.

### Analysis of AS features

Intron sequences with different AS types were extracted according to the coordinates reported by ASTALAVISTA. Transcript isoforms were translated based on the longest ORF. AS events were classified as 5'UTR, ORF and 3'UTR location according to their positions in different mRNA features. Perl scripts were written for analysis including AT content and changed nucleotides. The length of changed nucleotides was computed by comparing the alternative introns from two isoforms in a single AS event. For IR and ES, it was the length of the retained intron and skipped exon. For AltA, AltD and AltP, the length corresponded to the altered nucleotides that were present in the exon of one isoform, but included in the intron of the other isoform. The Pfam 27.0 database was searched to identify the domains affected by AS, with an E-value cutoff of 1e-10. The genes with AS modified domains were examined for their function annotations (on the basis of information in Phytozome) and classified into different biological processes. To determine significantly enriched protein domains associated with the AS occurrence, Fisher's exact test was conducted with a *p*-value cutoff of less than 0.05.

### Conservation analysis of AS-occurring genes

Orthology of the AS-occurring genes in *Populus* and *Eucalyptus* was investigated by the BLAST program (v2.2.21) with parameters of E-value < 1e-50, identity > 0.4 and coverage > 0.6. Expression patterns of the orthologous genes in *Populus* were established by microarray data from the NCBI Gene Expression Omnibus (GEO) database under accession numbers GSE30507 and GSE13990. Robust Multiarray Averaging (RMA) method was used to normalize microarray data across different tissues in Bioconductor according to previously described methods [53]. Enriched genes were defined as those with the highest expression level in xylem, compared to other tissues. The other genes were included as expressed genes. Heatmap.2 in R (v2.15.1) program was used to generate heatmaps of enriched expressed genes. *Populus* genes were converted to their orthologous genes in *Arabidopsis* and then used for GO annotation by agriGO [54]. Plant GOslim database and Fisher's exact test were implemented to identify enriched GO processes (FDR < 0.001).

### Identification of conserved AS events

Similar to previous reports [24, 32], conserved AS events in the two species were defined mainly by identification of the conserved introns in orthologous AS genes. To get conserved introns, the introns flanked by homologous exonic sequences were searched. In every AS event from *Populus* and *Eucalyptus*, 100 bp exonic sequences flanking the alternative introns were extracted. The tblastx program was used to search homologous exonic sequences across the two species with an E-value cutoff of 1e-5. If both of the flanking exonic sequences of a *Populus* intron were homologous to that of a *Eucalyptus* intron, the alternative introns were regarded as conserved introns. If the conserved introns were present in orthologous genes with the same AS type, the pair of AS event was considered a conserved AS event.

### Subcellular localization and RT-PCR analysis

Coding sequences of *PtIAA8* and *PtIAA8*^*IR*^ were cloned into pCAMBIA2300 backbone fused in frame with GFP under the control of the CaMV 35S promoter to determine their subcellular location. Tobacco leaves were transformed with constructs mediated with *Agrobacterium* strain GV3101 according to previous descriptions [55]. GFP fused proteins were examined with a confocal microscope (LSM 510 META; Zeiss) using 488 nm for excitation and 505 to 555 nm for emitted light capture. For RT-PCR, the xylem of *Populus* (*P. × euramericana*) and *Eucalyptus* (*E. grandis*) was used for RNA extraction. 500 ng RNA was reverse transcribed into cDNA by PrimeScript RT Master Mix (TaKaRa, Dalian, China) according to the manufacturer's protocols. Primers that span alternative introns were designed to perform PCR reactions in each species (Additional file 13).

### Availability of supporting data

All sequencing data were deposited in the Short Read Archive at NCBI under accession number SRA108028.

## Electronic supplementary material

Additional file 1: **Summary of reads mapping and transcript assembly.** (TIFF 233 KB)

Additional file 2:: **Assembled transcripts in** ***Populus*** **and** ***Eucalyptus.*** (XLS 6 MB)

Additional file 3: **AS events in** ***Populus*** **and** ***Eucalyptus.*** (XLS 2 MB)

Additional file 4: **Functional influences of AS events in different isoform ratios.** (TIFF 254 KB)

Additional file 5: **Confirmation of AS events by RT-PCR.** (TIFF 209 KB)

Additional file 6: **Nucleotide conservation at the junction sites of xylem expressed genes.** (TIFF 117 KB)

Additional file 7: **Changed nucleotides in different types of alternative splicing.** (TIFF 94 KB)

Additional file 8: **AS modified domains in** ***Populus*** **and** ***Eucalyptus.*** (XLS 1 MB)

Additional file 9: **Domain enrichment in AS genes.** (XLS 132 KB)

Additional file 10: **AS modified domains in wood formation-related genes.** (XLS 93 KB)

Additional file 11: **Orthologous groups of AS-occurring genes.** (XLS 156 KB)

Additional file 12: **Conserved alternative splicing events in** ***Populus*** **and** ***Eucalyptus.*** (XLS 52 KB)

Additional file 13: **Primers designed for RT-PCR confirmation.** (XLS 26 KB)
