# Supplementary figures and images for: Conservation and functional influence of alternative splicing in wood formation of Populus and Eucalyptus
Source: BMC Genomics. 2014 Sep 10;15(1):780. doi: 10.1186/1471-2164-15-780 (PMC4287496; doi:10.1186/1471-2164-15-780)

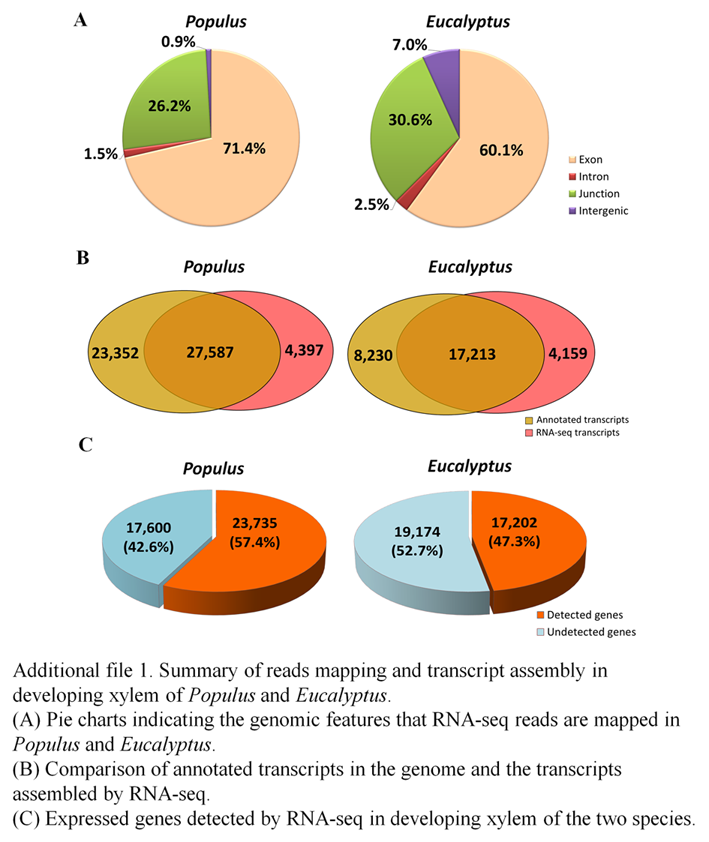

Supplement: Supplementary file 1 — Additional file 1: Summary of reads mapping and transcript assembly. (TIFF 233 KB) [file 12864_2014_6779_MOESM1_ESM.tiff]

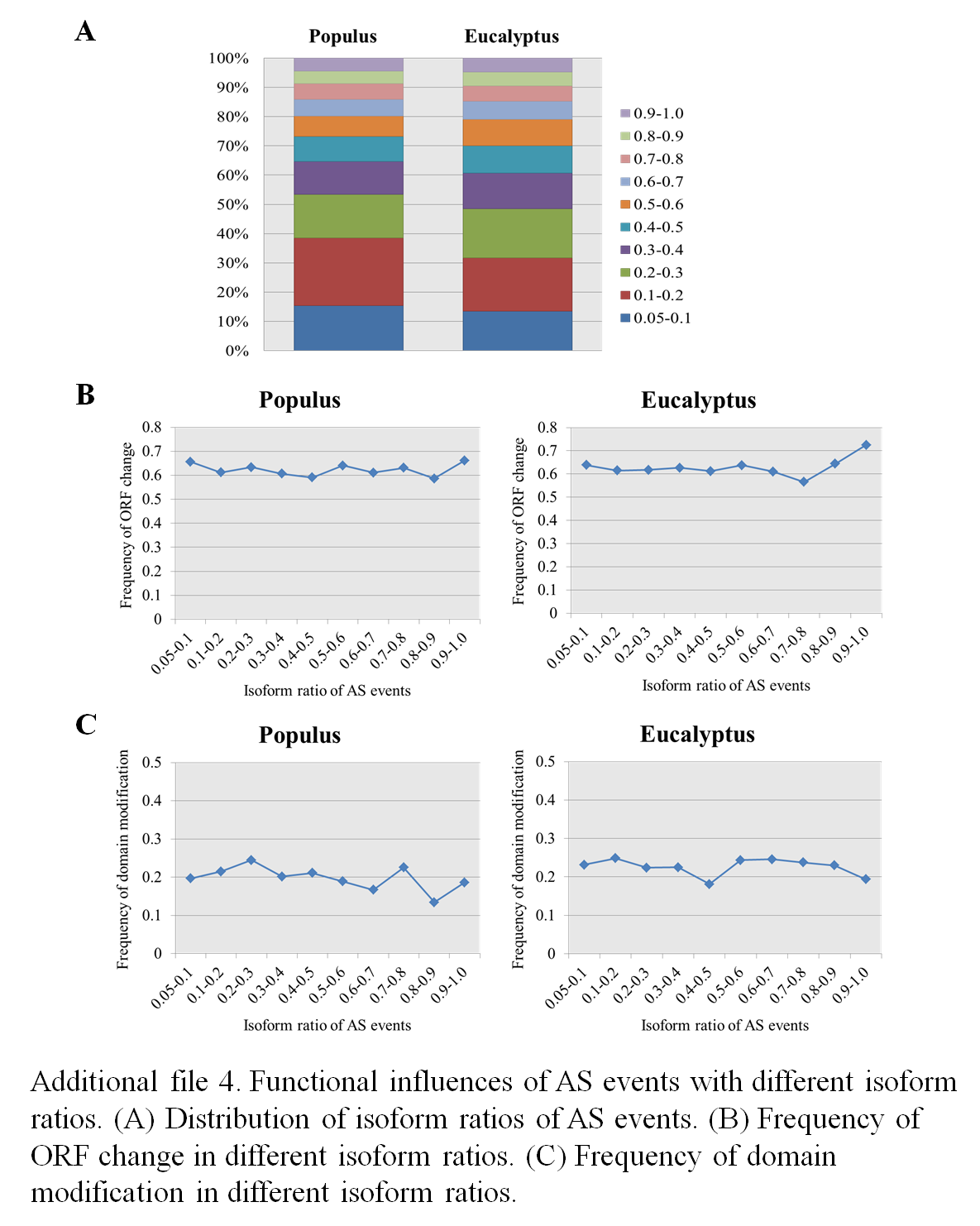

Supplement: Supplementary file 4 — Additional file 4: Functional influences of AS events in different isoform ratios. (TIFF 254 KB) [file 12864_2014_6779_MOESM4_ESM.tiff]

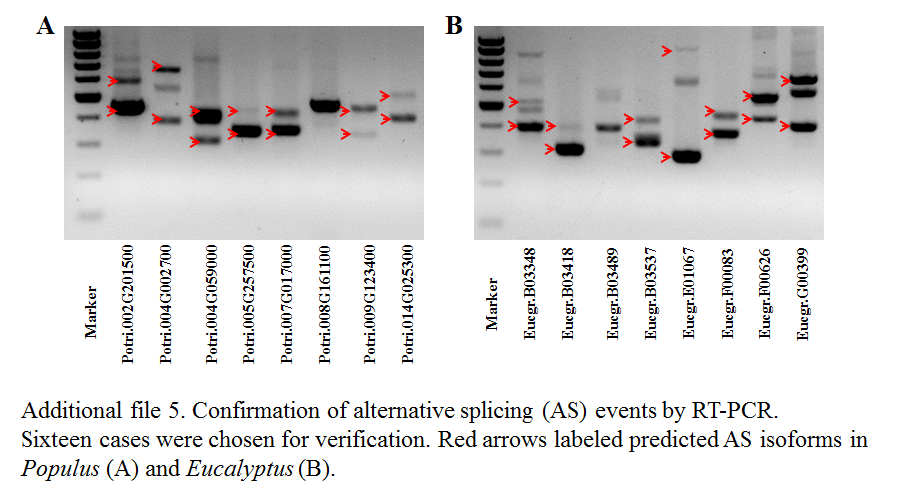

Supplement: Supplementary file 5 — Additional file 5: Confirmation of AS events by RT-PCR. (TIFF 209 KB) [file 12864_2014_6779_MOESM5_ESM.tiff]

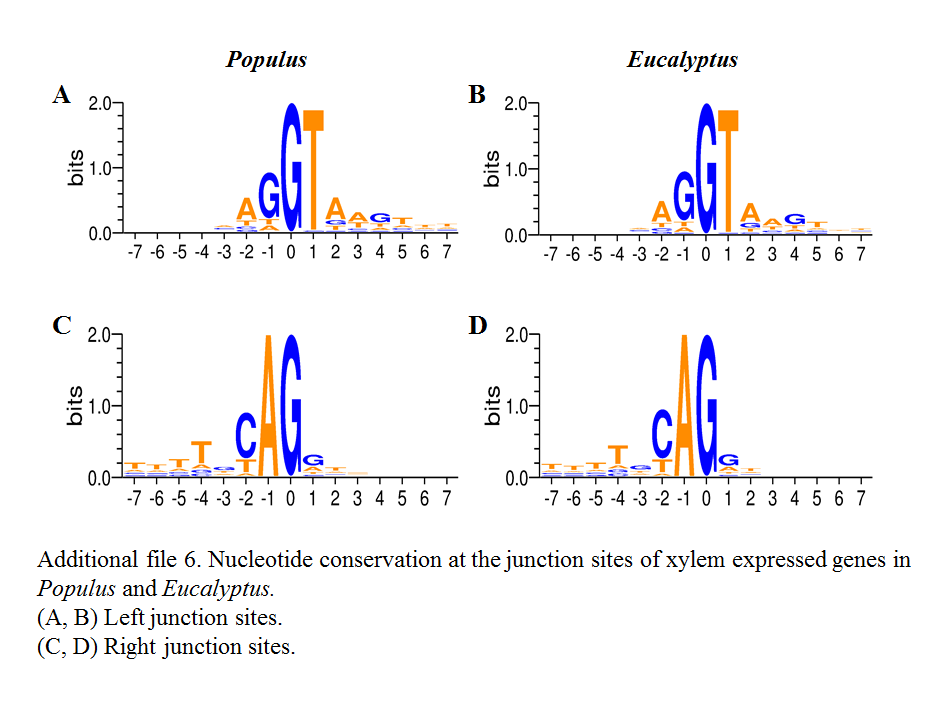

Supplement: Supplementary file 6 — Additional file 6: Nucleotide conservation at the junction sites of xylem expressed genes. (TIFF 117 KB) [file 12864_2014_6779_MOESM6_ESM.tiff]

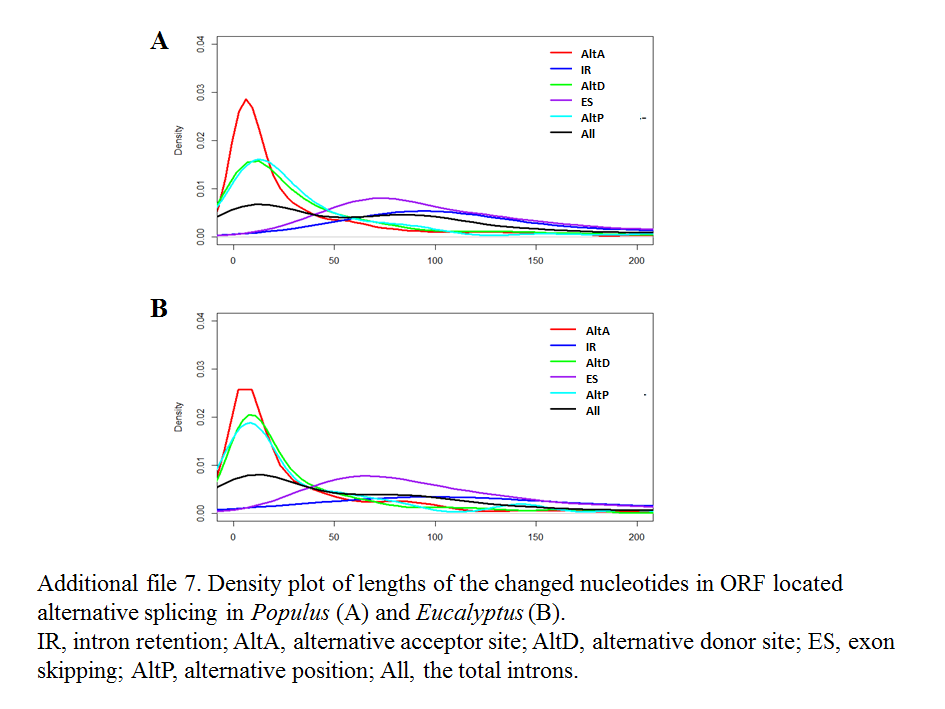

Supplement: Supplementary file 7 — Additional file 7: Changed nucleotides in different types of alternative splicing. (TIFF 94 KB) [file 12864_2014_6779_MOESM7_ESM.tiff]
